# Silk fibroin scaffolds: A promising candidate for bone regeneration

**DOI:** 10.3389/fbioe.2022.1054379

**Published:** 2022-11-25

**Authors:** Hao Wu, Kaili Lin, Cancan Zhao, Xudong Wang

**Affiliations:** ^1^ Department of Oral and Cranio-Maxillofacial Surgery, Shanghai Ninth People’s Hospital, Shanghai Jiao Tong University School of Medicine, Shanghai, China; ^2^ College of Stomatology, Shanghai Jiao Tong University, Shanghai, China; ^3^ Shanghai Key Laboratory of Stomatology, National Center for Stomatology, National Clinical Research Center for Oral Diseases, Shanghai, China; ^4^ Research Unit of Oral and Maxillofacial Regenerative Medicine, Chinese Academy of Medical Sciences, Shanghai, China

**Keywords:** silk fibroin (SF), bone regeneration, scaffolds, biocompatibility, biodegradability, osteogenesis

## Abstract

It remains a big challenge in clinical practice to repair large-sized bone defects and many factors limit the application of autografts and allografts, The application of exogenous scaffolds is an alternate strategy for bone regeneration, among which the silk fibroin (SF) scaffold is a promising candidate. Due to the advantages of excellent biocompatibility, satisfying mechanical property, controllable biodegradability and structural adjustability, SF scaffolds exhibit great potential in bone regeneration with the help of well-designed structures, bioactive components and functional surface modification. This review will summarize the cell and tissue interaction with SF scaffolds, techniques to fabricate SF-based scaffolds and modifications of SF scaffolds to enhance osteogenesis, which will provide a deep and comprehensive insight into SF scaffolds and inspire the design and fabrication of novel SF scaffolds for superior osteogenic performance. However, there still needs more comprehensive efforts to promote better clinical translation of SF scaffolds, including more experiments in big animal models and clinical trials. Furthermore, deeper investigations are also in demand to reveal the degradation and clearing mechanisms of SF scaffolds and evaluate the influence of degradation products.

## 1 Introduction

Caused by trauma, tumor and other pathological factors, bone defects can lead to dysfunctions and destruction of the musculoskeletal system (Burger et al., 2022). It remains challenging to achieve ideal bone regeneration in clinical practice (Lee et al., 2022; Mirkhalaf et al., 2022). Autografts and allografts are commonly applied to repair defected bones, but many factors limited their applications (Kundu et al., 2013; Guo et al., 2021). The application of exogenous scaffolds as bone substitutes seems an alternate strategy. The ideal scaffolds for bone regeneration should provide a biomimicking microenvironment to promote desirable cellular responses, which possess excellent biocompatibility, optimized mechanical properties, desirable morphology and structure. The desired biodegradability with safe by-products and controllable diffusion is also important (Zhang and King, 2020).

**FIGURE 1:**
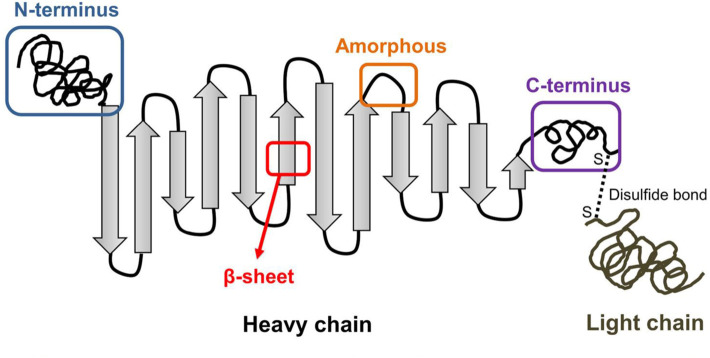
Illustration of SF structure including the heavy chain and light chain. (Wongpinyochit et al., 2018). Copyright 2018 American Chemical Society.

Among plenty scaffolds, silk fibroin (SF) is a promising candidate with numerous researches (Wenhao et al., 2020; Shen et al., 2022). In the structure of SF, the light (L) chains, heavy (H) chains and the hydrophobically linked glycoprotein P25 are crosslinked to form an H-L complex with anti-parallel beta-sheets (β-sheets) (Figure 1) (Wongpinyochit et al., 2018; Zuluaga-Velez et al., 2021). SF scaffolds have many advantages, including excellent biocompatibility, satisfying mechanical property, controllable biodegradability and structural adjustability (Zhang et al., 2021; Zuluaga-Velez et al., 2021; Li and Sun, 2022). Compared with biodegradable synthetic polymers such as poly (lactic acid) (PLA), SF scaffolds exhibit better biocompatibility and cell adhesion performance (Wenk et al., 2011). Besides, due to the formation of *ß*-sheets, SF scaffolds have better mechanical properties than collagens and chitosan (CS), with ultimate tensile strength of 300–740 MPa (Koh et al., 2015; Wang et al., 2020).

However, compared with native bone tissue, the mechanical properties of pure SF scaffolds are still insufficient (Melke et al., 2016; Soundarya et al., 2018). Further improvement is in demand to achieve better osteogenic capacity (Wu et al., 2021). Due to outstanding tunability, SF can be modified into different formats for certain applications, such as films, hydrogels and porous structures (Bakhshandeh et al., 2021). Besides, different organic and inorganic components can be mixed with pure SF to fabricate hybrid scaffolds to improve mechanical and biological performance (Wu et al., 2021). Another important modification method is surface modification, including physical modification, chemical modification and surface functionalization by bioactive components, which can make as-prepared SF scaffolds more bioactive (Hardy et al., 2018; Wang et al., 2019; Moses and Mandal, 2022).

Despite the large number of related researches, there still lack deep investigations on the long-term *in vivo* safety of SF scaffolds, which is closely related to the degradation products. The studies on degradation and cleaning mechanism of SF scaffolds are also far from satisfactory. It can be helpful to deeply understand the *in vivo* interaction of SF scaffolds with hosts’ tissues to better inspire further modifications. Moreover, among numerous fabrication techniques and modification methods, it can be difficult to make a suitable choice for different applications, where a comprehensive classification and summary can be helpful.

**FIGURE 2:**
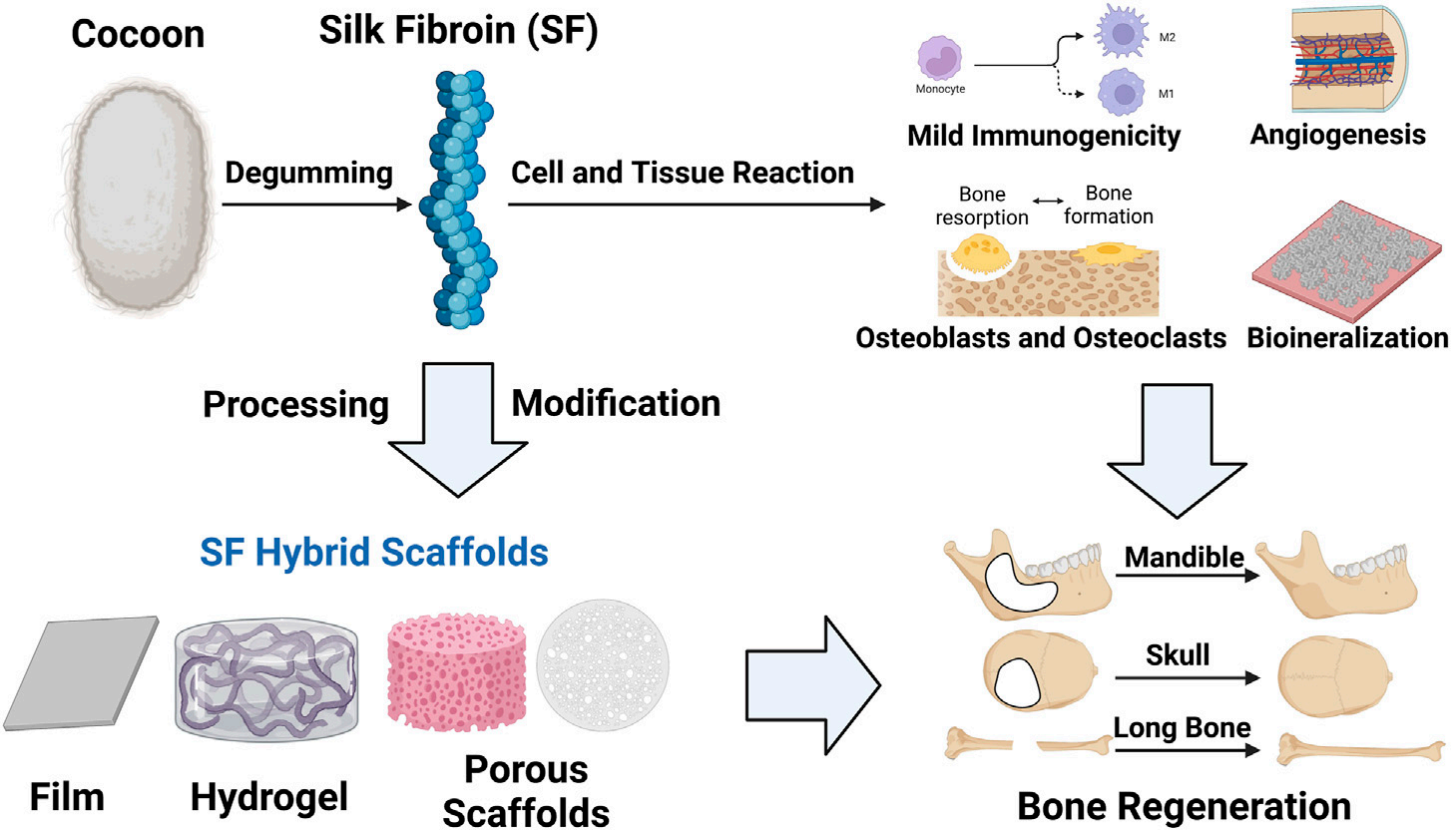
Graphical diagram for fabrication of SF scaffolds and their cell and tissue responses, which makes contributions to bone regeneration. (Created with BioRender.com).

Herein, we summarize the cell and tissue interaction with SF scaffolds, the fabrication techniques and modifications for better bone regeneration (Figure 2). We hope to give a deep insight into the SF scaffolds applied in bone regeneration and inspire research enthusiasm for better development and improvement of SF scaffolds.

## 2 Cell and tissue interaction with Silk fibroin scaffolds

As a highly dynamic vascularized tissue, bone tissue consists of cellular components, extracellular matrix (ECM) and minerals. Through achieving a deeper understanding of SF’s interaction with cellular and non-cellular components, the design of advanced SF-based scaffolds can be better inspired.

### 2.1 Immune response

Silk materials have been proved biocompatible with long history of application, but some rarely-happened adverse immunological events cannot be ruled out, which may be caused by the presence of silk sericin (SS) proteins. Wang et al. (Wang et al., 2020) combined SF and SS in different mass ratios and detected the immune responses caused by different scaffolds. The results showed that the macrophages were activated by the addition of SS and secreted more proinflammation (M1)-related cytokines. The SF itself showed satisfying biocompatibility with higher antiinflammation (M2) phenotype ratio of macrophages. As for *in vivo* evaluation, Gorenkova et al. (Gorenkova et al., 2021) evaluated the immune response of self-assembled SF hydrogels *via* Balb/c mice models. The inflammatory response was comparable or even lower than the benchmark material, polyethylene glycol (PEG), which did not activate tissue regeneration and served as the baseline marker for tissue responses. Overall, these studies demonstrate that the SF scaffolds can exhibit favorable biocompatibility after degumming and sterilization.

Despite these encouraging researches, there still exist problems about long term *in vivo* safety of SF scaffolds and long-term immune responses need further investigation to achieve better understanding. The immune response to degradation products of SF scaffolds should also be considered, where the production of proinflammatory cytokine and phagocytosis may be induced by fractions of SF fibers (Gellynck et al., 2008). Lundmark et al. (Lundmark et al., 2005) found that the degradation products of SF could cause amyloidogenesis and tissue degeneration. Therefore, it is necessary to carry out long-term studies of SF scaffolds on their degradation products.

### 2.2 Osteogenic cellular response

Meinel et al. (2005) claimed that the plain silk scaffolds could only provide an appropriate environment for cellular proliferation, where the ingrown cells showed poor osteogenic differentiation. However, later researches demonstrated that SF could activate expression of osteogenesis-related genes, including alkaline phosphatase (ALP), Runt-related transcription factor 2 (Runx2), collagen I (COL I), osterix, osteocalcin (OCN) and CD29/CD44 (Miyamoto et al., 2013; Panda et al., 2015). Jung et al. (2013) found the SF could serve as the suppressor of the Notch pathway, which could down-regulate of osteogenesis and thus promote osteogenesis. The amide groups and high *ß*-sheet contents of SF could also induce osteogenic differentiation, where the *ß*-sheet structure could provide a stiff matrix environment for osteoblasts.

However, considering results of other *in vivo* experiments, plain SF scaffolds have insufficient ability to completely regenerate large bone defects (Mottaghitalab et al., 2015). Uebersax et al. (2013) implanted two different SF scaffolds into the long bones of sheep and found the poor bone formation in both scaffolds. Song et al. (2011) applied SF film to repair rabbit calvarial defects (φ 8 mm), and found the calvarial defects actually failed to completely recover after 8 weeks. Notably, when pre-seeded with undifferentiated stem cells before implanted, the capability of bone formation can be significantly improved with pre-differentiated cells (Park et al., 2015; Sartika et al., 2020). Considering the long-term procedure of autologous cell isolation and culture, the cell-free SF scaffold is still a better choice.

### 2.3 Bone resorption

During bone regeneration, it is important to take bone homeostasis into consideration, which is maintained by the balance of dynamic bone formation and resorption. Osteoblasts originate from multipotent mesenchymal stem cells (MSCs) and secrete the osteoid matrix. Osteoclasts arise from the mononuclear cell lineage and are responsible for bone matrix resorption (Borciani et al., 2020).

However, so far, only a few literatures are focused on interaction between SF scaffolds and osteoclasts. Jones et al. (Jones et al., 2009) cultured murine osteoblasts and osteoclasts on SF scaffolds. When monocytes were cultured separately, cells aggregated together and the expression of tartrate resistant acid phosphatase (TRAP) was positive, as the osteoclast marker. However, when co-cultured with osteoblasts, individual TRAP positive cells spread evenly amongst osteoblasts, forming a homogeneous layer. Furthermore, Chon et al. (Chon et al., 2012) reported that SF hydrolysate could inhibit RANKL-induced TRAP formation, osteoclast-related gene expression and signaling pathways in RAW 264.7 cells. Meanwhile, the SF hydrolysate could induce apoptosis signaling cascades of osteoclasts. On this basis, SF scaffolds seem an ideal choice for bone regeneration to repair bone defects with its capacity to inhibit activity of osteoclasts. However, more researches should be carried out in this field to further understand the effect of SF scaffolds on osteoclasts and bone remodeling.

### 2.4 Vascular ingrowth

The vascular ingrowth is another important factor of bone regeneration, which can improve oxygen and nutrient diffusion (Li et al., 2021). It also influences the differentiation of MSCs into osteoblasts and osteoid formation (Yan et al., 2019; Song et al., 2020). The enhanced osteogenic differentiation promotes the secretion of soluble factors, such as bone morphogenetic protein-2 (BMP-2) and beta-catenin, and in turn benefits the vascularization process (Niu et al., 2019). During the biomineralization period, it is also helpful to form functional vascular networks and thus achieve desired bone formation.

The mild inflammatory response can induce the vascular growth and the ingrowth of vessels into SF scaffolds (Thurber et al., 2015; Rameshbabu et al., 2020). Usually, SF scaffolds used for bone regeneration exhibit high porosity and thus allow the vessel ingrowth, which can be improved by pre-seeded cells before implantations. Watchararot et al. (2021) seeded human adipose-derived stem cells (hADSC) onto SF scaffolds and implanted them into chick chorioallantoic membrane with the pore diameter of 513.96 ± 4.99 μm and porosity of 77.34 ± 6.96%. A capillary network of spoke-wheel pattern was induced by the seeded scaffolds 3 weeks earlier than unseeded scaffolds, indicating an early angiogenesis. Sun et al. (Sun et al., 2016) also found the co-cultural of endothelial cells and human mesenchymal stem cells (hMSCs) could significantly improve angiogenesis of SF scaffolds which might result from vascular endothelial growth factor (VEGF) and other angiogenic factors secreted by the pre-seeded cells.

### 2.5 Matrix mineralization

Biomineralization of bone ECM is highly dynamic but well-regulated to obtain diverse organic-inorganic hybrid structures (Kundu et al., 2020). Native bone ECM exhibits three-dimensional (3D) structure with porous morphology and organic-inorganic components. The different physiologic conditions and ECM compositions also influence the deposition of hydroxyapatite (HAP) nanocrystals (Thrivikraman et al., 2019). The mineralized matrix can in turn induce osteogenic differentiation and the formation of mature bone tissues (Xiong et al., 2011). Therefore, an ideal bone graft should have capability to induce biomineralization, similar to natural bone ECM.

SF scaffolds have been proved able to improve the deposition of HAP nanocrystals in simulated body fluid (SBF) solution (Zaharia et al., 2012; Nourmohammadi et al., 2017; Kundu et al., 2020). The amorphous spacers can serve as nucleation sites and promote the deposition of HAP crystals, which is similar to the role of Col I in natural bone (Marelli et al., 2012; Jin et al., 2015; Vetsch et al., 2015). After pretreatment, the formation of HAP crystallization can be facilitated by the electrostatic interaction between the functional groups and calcium ions (Ca^2+^) (Choi et al., 2012). Huang et al. (Huang et al., 2021) fabricated organized SF film and study the deposition of amorphous calcium phosphate (CaP) in phosphate buffer saline (PBS) and enzyme solution, where a mixture of tricalcium phosphate (TCP) and HAP crystals formed in PBS solution but only HAP crystals could be observed in enzyme solution. The difference possibly resulted from the degradation of SF induced by enzyme, which led to different SF contents and solution pH environment. Meanwhile, it is also interesting to find SF scaffolds from different sources have different performance in biomineralization process. Zhang et al. (Zhang et al., 2020) compared the effect of A*ntheraea pernyi* SF and *Bombyx mori* SF fibers as templates of biomineralization and found the *Antheraea pernyi* SF could induce better mineralization, which resulted from more acidic amino acids of hydrophilic amorphous fractions. Sahu et al. (2015) also found nonmulberry SF scaffolds had better osteoconductivity and biomineralization performance than mulberry SF scaffolds.

## 3 Techniques to fabricate Silk fibroin hybrid scaffolds

The techniques to fabricate different SF-based scaffolds depend on the formats they appeared in bone regeneration, such as films, hydrogels and porous scaffolds, which have been summarized in Table 1.

**TABLE 1:** Summary of fabrication techniques of SF scaffolds for bone regeneration.

Scaffold type	Silk source	Fabrication techniques	Bioactive components	References
Mat	*Bombyx mori*	Electrospinning	Calcium zinc silicate	Hadisi et al. (2020)
Mat	*Bombyx mori*	Electrospinning	HAP	Valarmathi and Sumathi, (2020)
Mat	*Bombyx mori*	Electrospinning	Laponite	Atrian et al. (2019)
Mat	*Bombyx mori*	Electrospinning	Bioactive glass (BG) and HAP	Liu et al. (2019)
Sponge	*Bombyx mori*	Freeze drying	Alumina	Zafar et al. (2020)
Sponge	*Bombyx mori*	Freeze drying	HAP	Nie et al. (2019)
Sponge	*Bombyx mori*	Glycerol crosslinking and directional field freeze technology	HAP and graphene oxide (GO)	Wang et al. (2020b)
Sponge	*Bombyx mori*	EDC/NHS click-chemistry method	HAP, carboxymethyl CS, cellulose nanocrystals and strontium	Zhang et al. (2019)
Sponge	*Bombyx mori*	Glutaraldehyde crosslinking and freeze drying	CS and magnetite	Aliramaji et al. (2017)
Sponge	*Bombyx mori*	Freeze drying	Titanium dioxide (TiO_2_)	Johari et al. (2017)
Sponge	*Bombyx mori*	Freeze drying	Fluoridated TiO_2_	Johari et al. (2018a)
Sponge	*Bombyx mori*	Freeze drying	TiO_2_	Johari et al. (2018b)
Sponge	*Bombyx mori*	HRP-crosslinking, salt leaching and freeze drying	β-TCP	Ribeiro et al. (2019)
Hydrogel	*Bombyx mori*	Sonication and crosslinking	Vancomycin and halloysite	Avani et al. (2020)
Hydrogel	*Bombyx mori*	Heating and crosslinking	DFO and HAP	Wang et al. (2021)
Hydrogel	*Bombyx mori and Antheraea assama*	HRP-crosslinking	HAP and strontium	Moses et al. (2020)
Hydrogel	*Bombyx mori*	Free radical polymerization technique	Magnetite	Tanasa et al. (2020)
Hydrogel	*Bombyx mori*	3D bioprinting	Gelatin, riboflavin, articular cartilage-derived progenitor cells (ACPCs), dental pulp derived stem cells (DPSCs), hMSCs.	Piluso et al. (2020)
Hydrogel	*Bombyx mori*	3D bioprinting	Tris (2,2′-bipyridyl) dichlororuthenium hexahydrate (Ru) and sodium persulfate (SPS), Human articular chondrocytes (HACs)	Cui et al. (2020)
Hydrogel	*Bombyx mori*	Photo-initiated crosslinking	Ru, SPS,	Bap tista et al. (2020)
Film	*Bombyx mori*	*In situ* co-precipitation	HAP	Mobika et al. (2020)
Film	*Bombyx mori*	*In situ* co-precipitation	Chlorin e6	Huang et al. (2022)
Film	Not mentioned	Plasma splashing procedure	CS, polyethylene terephthalate, HAP	Jiang et al. (2020)

### 3.1 Silk fibroin hybrid films

On basis of deposition techniques and drying processes, the technology to fabricate SF films has been well developed, including *in-situ* co-precipitation method (Mobika et al., 2020; Huang et al., 2022), plasma splashing procedure (Jiang et al., 2020) and matrix-assisted pulsed laser evaporation (Miroiu et al., 2010). To achieve better functionalization, during film preparation, external components can be added, like HAP, silver (Ag) and other bioactive factors, which makes SF hybrid films multifunctional and thus further enhance osteogenesis, including better mechanical properties, angiogenesis, antiinflammation, antibacterial capacity and so on. Jabbari et al. (Jabbari et al., 2019) developed a CS/SF hybrid film *via* solvent casting method, loading reduced graphene oxide (rGO). With the addition of rGO, the swelling ratio and conformability of hybrid film was accordingly increased, and the evaluation of ALP activity and alizarin red staining also demonstrated the promoted osteogenic performance.

### 3.2 Silk fibroin hybrid hydrogels

The cross-linking techniques are widely used to fabricate SF hydrogels, including mechanical cross-linking, chemical crosslinking, enzymatic crosslinking and light triggered crosslinking techniques. The mechanical cross-linking technique is simple and economical *via* ultrasound pulses or temperature changes. As for chemical cross-linking, enzymatic crosslinking and light-triggered crosslinking techniques, external components should be introduced as initiators to achieve better crosslinking. For example, the horseradish peroxidase (HRP) cross-linking technique can control the sol-gel transition better and increase the content of *ß*-sheets (Moses et al., 2020; Zuluaga-Velez et al., 2021). Furthermore, immersing SF in methanol or ethanol can transform α-helix structure into *ß*-sheet structure and thus improve the mechanical properties of as-prepared SF hydrogels (Johari et al., 2018a). Piluso et al. (Piluso et al., 2020) developed a rapid riboflavin-mediated crosslinking technique to fabricate cytocompatible SF hydrogel with different riboflavin/sodium persulfate (Ru/SPS) ratio, which exhibited a viability over 80% for all cell types.

With the development of addictive manufacture, 3D printing has become an efficient on-demand manufacturing technique for SF hydrogel, which can help create individual scaffolds of both small and large scale. Furthermore, the cell-laden 3D bioprinting technique permits living cells and biomaterials to be placed in highly organized scaffolds to form complex structures for different applications, which is one of the latest trends in regenerative medicine (Buitrago et al., 2018). Sharma et al. (Sharma et al., 2019) used 3D bioprinting to fabricate a hBMSCs-laden SF/gelatin/CaCl_2_ hybrid hydrogel with sustained release of Ca^2+^ and increased *ß*-sheet contents, which facilitated the osteogenic differentiation and mineralization of the hBMSCs through Wnt/β-catenin pathway. However, due to the formation of *ß*-sheet, the increase of stiffness and crystallinity can make SF hydrogels brittle and hard to be remodeled by cells (Partlow et al., 2014). In order to overcome the challenges for cell encapsulation, different alternative crosslinking techniques have been explored for cell-laden SF hydrogels, such as chemical crosslinking, enzymatic crosslinking and other redox-based crosslinking, among which photo-triggered crosslinking technique exhibit great potential (Raia et al., 2017; Cui et al., 2020). Cui et al. (Cui et al., 2020) developed a rapid photoredox crosslinking technique to fabricate SF hydrogel, which allowed high cell densities (15 million cells/mL) for cell encapsulation and retained high cell viability (>80%) simultaneously. The photocrosslinked SF hydrogel could reduce spontaneous transition to *ß*-sheet and possess more stable mechanical properties, demonstrating the immense potential of this crosslinking system for biofabrication and tissue regeneration.

### 3.3 Silk fibroin hybrid porous structures

Porous SF scaffolds include flat structures and sponge-like structures. Flat structures can be formed by fibers and exhibit porous characteristic. Almost all the reported fibers are fabricated by electrospinning, and only a few are achieved by knitting machines (Shi et al., 2013; Biagiotti et al., 2022) or spray-drying/pressing (Dong et al., 2020). Sponge-like structures are the most common reported format as porous scaffolds, showing 3D porosities (Hollister, 2005). Sponge-like structures can be achieved by directional temperature field freezing technology (Wang et al., 2020), salt leaching (Samal et al., 2015; Yan et al., 2015), sonication (Gholipourmalekabadi et al., 2015), 3D-printing (Jia et al., 2019), crosslinking (Teimouri et al., 2015; Aliramaji et al., 2017) and combination of layer-by-layer process and freeze drying (Jia et al., 2019). The pore sizes of freeze-dried sponges are below 100 μm, but they can be controlled by changing solvents, pH and temperature (Kundu et al., 2013). *Via* particle leaching, solvent casting or gas-foaming techniques, the pore structures can be better controlled (Harris et al., 1998).

## 4 Modifications of silk fibroin hybrid scaffolds for osteogenesis

The plain SF scaffolds exhibit limited osteogenic activity. Modifications should be carried out to enhance osteogenic activity of plain SF scaffolds. In this part, we classify different modification methods in to three kinds: composition adjustment, structure design and surface modification.

### 4.1 Composition adjustment of silk fibroin hybrid scaffolds

#### 4.1.1 Silk fibroin/inorganics hybrid scaffolds

The bioactive inorganic components applied to form SF/inorganics hybrid biomaterials include CaP (Gupta et al., 2016; Shao et al., 2016; Ribeiro et al., 2018), graphene oxide (GO) (Balu et al., 2018; Wang et al., 2018), titanium dioxide (TiO_2_) (Pan et al., 2014; Johari et al., 2018a), silica (SiO_2_) (Mieszawska et al., 2010; Maleki et al., 2019) and bioactive glass (BG) (Bidgoli et al., 2019; Du et al., 2019).

**FIGURE 3:**
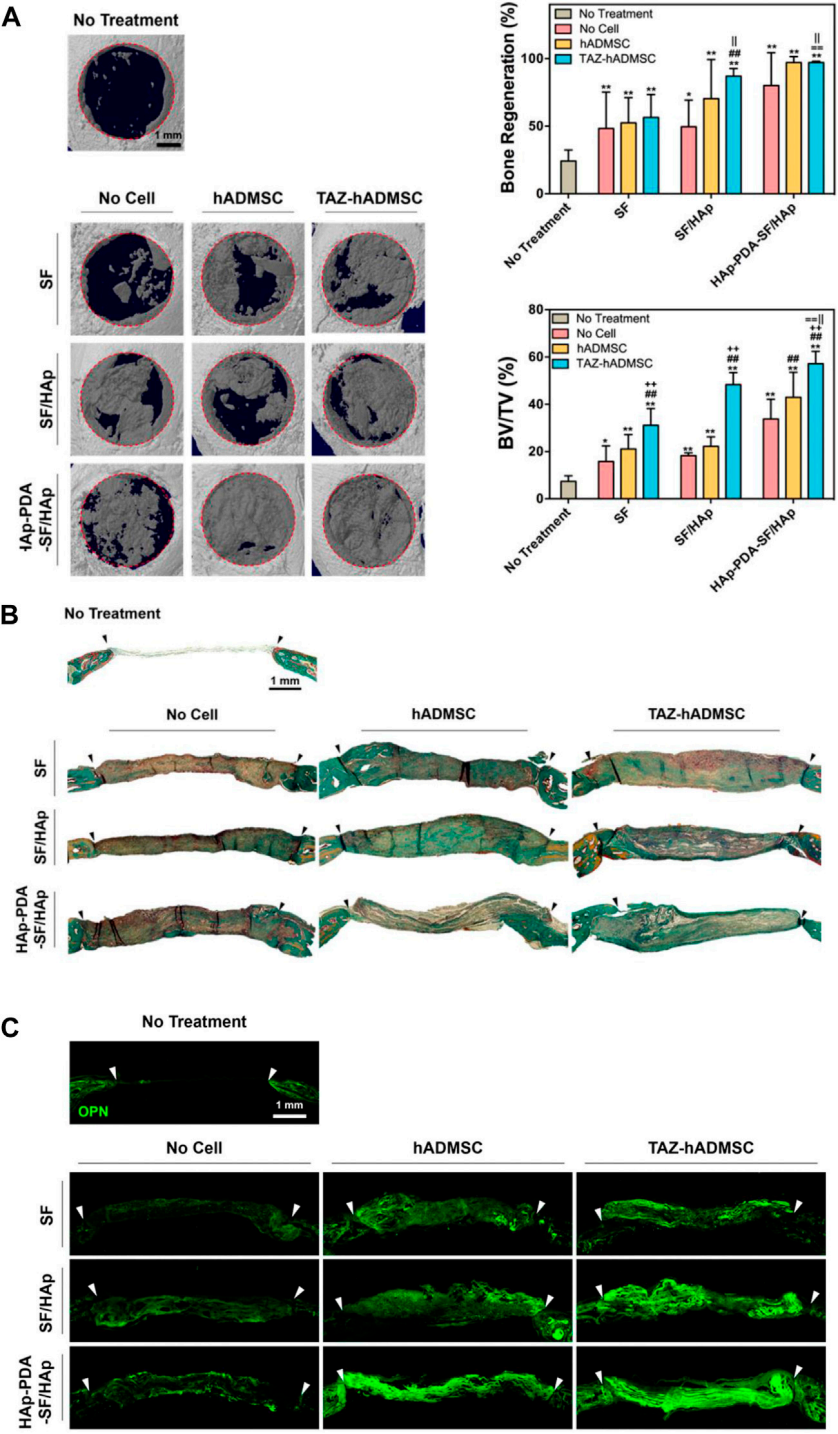
*In vivo* evaluation of the HAP-functionalized SF scaffolds. **(A)** 3D images of the regenerated calvarial defects treated by different scaffolds and quantitative evaluation. **(B)** Goldner’s trichrome staining of regenerated calvarial defects. **(C)** Osteopontin immunofluorescence staining of regenerated calvarial defects after different treatments. (Ko et al., 2018) Copyright 2018 American Chemical Society.

The CaP, SiO_2_, TiO_2,_ and BG components can be classified into ceramic biomaterials, exhibiting osteoconductivity, good compression and corrosion resistance, among which HAP has been most applied (Wu et al., 2022). HAP has similar structure and chemical composition with natural bone, which allows them to provide a biomimetic interface for facilitated osseointegration. Ko et al. (2018) integrated HAP with SF scaffolds *via* blending and alternate soaking, and the modulus was over two times of plain SF scaffolds, which also induced better osteogenic differentiation of human adipose-derived mesenchymal stem cells (hADMSCs) and bone regeneration of calvarial defects (Figure 3). SiO_2_ and BG are also promising for bone regeneration, whose degradation products show osteoconductive properties (Nikolova and Chavali, 2019). Combining TiO_2_ nanoparticles with SF scaffolds can facilitate scaffolds with better biocompatibility and osteoconductivity, where TiO_2_ can help SF hybrid scaffolds to mechanically interlock with bone tissues and promote cell attachment and proliferation (Johari et al., 2017). TiO_2_ can also enhance the mechanical properties of hybrid biomaterials *via* facilitating the formation of *ß*-sheet structure (Pan et al., 2014).

GO is one of graphene derivatives, showing good biocompatibility and osteogenic activities, which is another important inorganic component for SF modification. The great advantage to incorporate GO into SF scaffolds is the improvement of mechanical property (Balu et al., 2018). Moreover, SF/GO hybrid scaffolds also show improved biocompatibility and antibacterial property (Wang et al., 2018). Shuai et al. (Shuai et al., 2018) developed SF/GO matrix to evaluate its effect on stem cell fate, where the modulus increased with introduction of GO and the unique topography could promote early adhesion and osteogenic differentiation of hMSCs without additional inducers.

#### 4.1.2 Silk fibroin/organics hybrid scaffolds

The bioactive organic components applied to form SF/inorganics hybrid scaffolds include synthetic and natural polymers, such as polycaprolactone (PCL) (Bhattacharjee et al., 2016; Cengiz et al., 2019; Cengiz et al., 2020), collagen (Shi et al., 2017; Bandyopadhyay and Mandal, 2020), CS (Bissoyi et al., 2018), cellulose (Lee et al., 2013; Barud et al., 2015), alginate (ALG) (Zhang et al., 2015; Patil and Singh, 2019) and so on.

PCL is one of main synthetic polymers applied in SF hybrid scaffolds (Dong et al., 2021). The as-prepared scaffolds possess the osteoconductive effect of SF and osteogenic effect of PCL simultaneously, making it excellent for bone regeneration (Li et al., 2020). Bhattacharjee et al. (Bhattacharjee et al., 2016) fabricated SF/PCL nanofibers *via* electrospinning, where the tensile strength increased by about one fold and the osteogenic differentiation was enhanced compared with plain PCL scaffold.

Introduction of natural polymers seems a feasible approach to enhance cell attachment and osteogenesis due to their favorable biocompatibility and biofunctional components (Wang et al., 2016). SF/collagen and SF/gelatin hybrid scaffolds can exhibit excellent biocompatibility and ECM-mimicking structure, which is able to accelerate the bone formation (Bharadwaz and Jayasuriya, 2020; Wu et al., 2021). SF/CS scaffolds also have great potential in bone regeneration due to the bioactive RGD sequence (Bissoyi et al., 2018). Meanwhile, the combination of cellulose with SF can improve the biodegradability and provide more space for bone regeneration (Ni et al., 2020). Introducing ALG into SF scaffolds can be a promising approach to overcome the poor cell-adhesive property of ALG and the ALG can replace the role of gelatin with lower cost (Patil and Singh, 2019). Perteghella et al. (Perteghella et al., 2017) developed ALG/SF microcarriers with spherical geometry and average diameter of 400 μm, where MSCs adhered rapidly and preserved their potential of multi-lineage differentiation in this innovative 3D culture system.

### 4.2 Structural design of SF hybrid scaffolds

#### 4.2.1 Films

The favorable performance of SF film makes it a promising strategy for bone regeneration. Li et al. (2022) combined SS and SF to fabricate hybrid films and the breaking strength and breaking elongation increased significantly, which also exhibited faster and well-regulated HAP deposition rate than plain SF films. Furthermore, topographical pattering of SF films can help to achieve better osteogenic performance *in vivo*. Sayin et al. (2017) prepared collagen/SF hybrid films with microchannel patterns, and human osteoblasts and adipose-derived stem cells (ADSCs) were seeded and aligned on the ridges and in the grooves of the patterned film, where stimulate anisotropic osteogenesis was simulated.

Notably, SF film is also an effective barrier to avoid collapse of surrounding soft tissues into the defect cavity and ensure the successful bone repair. Smeets et al. (2017) evaluated three different SF films and commercial collagen membranes. Compare to collagens, the lower resorbability of SF membranes could promote bone regeneration for a longer period. Therefore, SF films are able to meet some special demands of bone regeneration.

#### 4.2.2 Porous scaffolds

Porous SF scaffolds have ideal structures for bone regeneration due to their great similarity to the *in vivo* microenvironment. Varkey et al. (2015) compared the impact of different SF structures on MG63 cell attachment and proliferation. The sponge structure showed the highest porosity of 67% and could maintain its structural integrity, where the secreted collagen also increased with culture time, demonstrating the increasing production of ECM. Correia et al. (2012) also fabricated SF porous scaffolds with different pore sizes to investigate their effects, where the SF scaffold with 400–600 μm pore sizes showed better HAP deposition and expression of osteogenic proteins, and further promoted bone tissue formation, demonstrating the importance of scaffold’s pore structures.

However, the mechanical properties of SF porous scaffolds are not satisfying, which limits its application in load-bearing locations. To obtain better mechanical and biological outcomes, external components have been incorporated with SF porous sponges (Kundu et al., 2013). Gupta et al. (2016) fabricated a biomimetic, osteoconductive tricomposite scaffold using HAP, SF fiber from *Antheraea assama* and its SF solution. The SF-reinforced tricomposite scaffolds exhibited about 5-fold higher compressive modulus and better osteogenic capacity. Therefore, SF-based biomimic porous scaffolds with tough mechanical properties are promising in application of bone regeneration. However, it is still insufficient for load-bearing bone and further investigations should be carried out.

#### 4.2.3 Hydrogels

**FIGURE 4:**
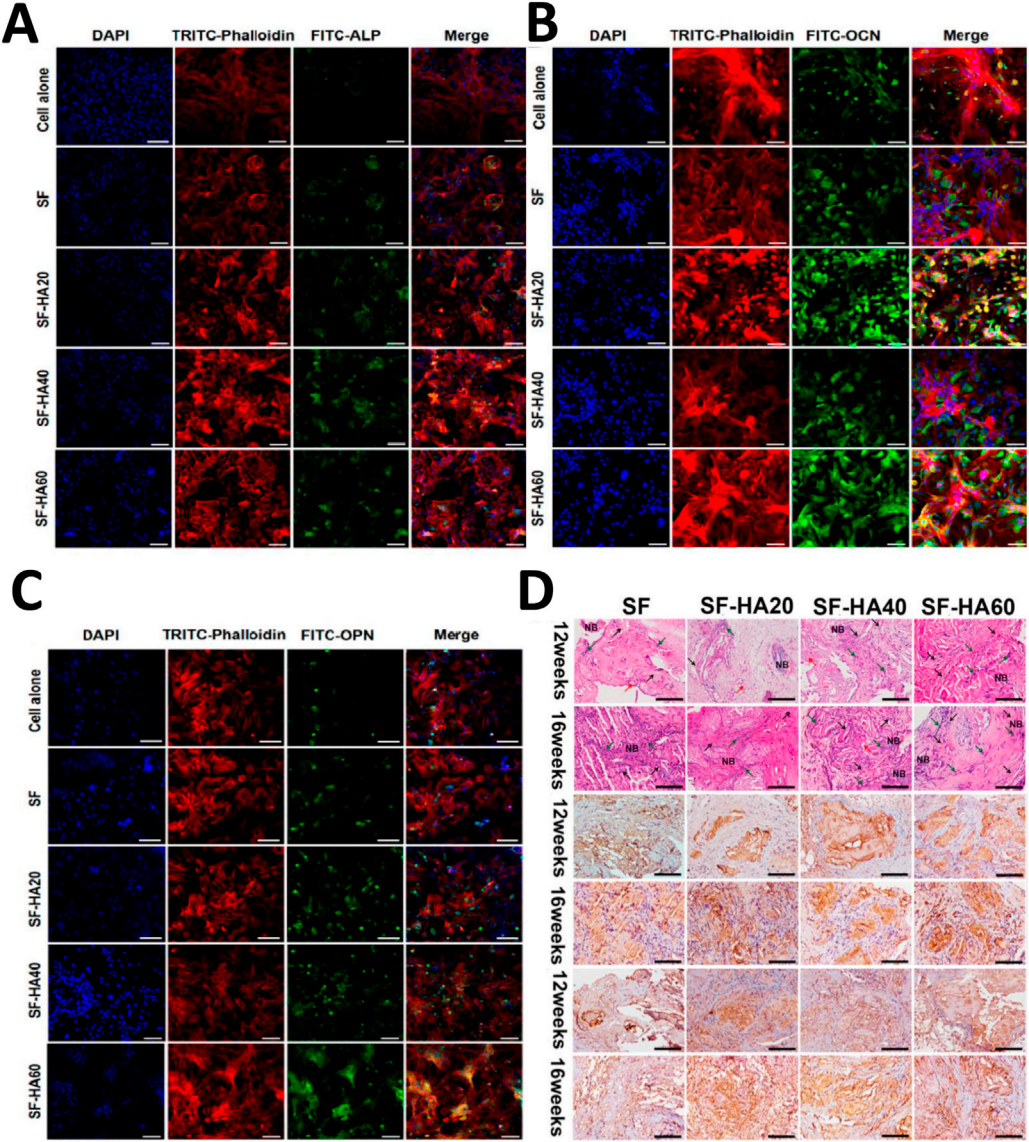
Immunofluorescence staining and histological analysis of osteogenic capacity *in vitro* and *in vivo*. **(A)** ALP expression after 7 days in different hydrogels. **(B)** OCN expression after 21 days in different hydrogels. **(C)** OPN expression after 21 days in different hydrogels. The nucleus was stained blue by DAPI and the F-actin was stained red by tetramethylrhodamine (TRITC) conjugated to phalloidin, while ALP, OCN, and OPN were stained green by antibodies conjugated with fluorescein isothiocyanate (FITC). **(D)** HE and immunohistochemistry staining of OCN and OPN in different hydrogels. (Ding et al., 2017) Copyright 2017 American Chemical Society.

Due to the similarity to microenvironmental of natural tissues, hydrogels have remarkable advantages in bone regeneration (Liaw et al., 2018). SF hydrogels have been widely developed *via* various crosslinking techniques. Ding et al. (2017) fabricated an injectable SF-based hydrogel *via* the combination of water-dispersible SF-HAP nanoparticles and the thixotropic SF nanofiber hydrogel. This nano-scale hydrogel system with homogeneously-distributed HAP nanoparticles showed good biocompatibility and osteogenesis *in vitro*, as well as better bone formation *in vivo* (Figure 4). Ribeiro et al. (2015) combined SF and nano HAP to develop hybrid hydrogels with favorable porosity, mechanical properties and osteoconductivity, which improved cellular metabolism and ALP activity of MG63 cells. These researches have proved the efficiency of SF hybrid hydrogels to induce bone regeneration.

Furthermore, SF hydrogels are ideal drug carriers. An injectable SF-based hybrid hydrogel prepared by Roohaniesfahani et al. (2019) exhibited the capacity to release bioactive silicon, strontium, and magnesium ions simultaneously to promote osteogenesis and angiogenesis. The seeded osteoblasts showed promoted cell proliferation, ALP activity, and enhanced osteogenic gene expression compared to plain SF hydrogel. The *in vivo* results exhibited decreased fibrous capsule formation and increased new blood vessels around the hydrogel. Besides, multiple SF hydrogels have been prepared to improve their osteogenic capacities with sustainable release of bioactive factors (Ding et al., 2019; Avani et al., 2020; Zarrin et al., 2022). Therefore, SF hydrogels exhibit significant advantages when serving as bioactive scaffolds for bone repair both as matrices and carriers.

### 4.3 Surface modification of SF hybrid scaffolds

#### 4.3.1 Physical modification

Physical modifications of SF scaffolds include ultraviolet (UV) treatment, gas treatment and plasma treatment (Lau et al., 2020). UV irradiation of SF scaffolds can increase wettability and improve cell adhesion without obvious weight loss, crystallinity change and strength decrease (Li et al., 2012; Khosravi et al., 2018). As a powerful oxidizing agent, ozone (O_3_) gas treatment can increase the pliability of SF scaffolds because of the oxidation of amino acid residues (Li et al., 2012). Plasma treatment of SF scaffolds by different working gases (SO_2_, NH_3_, and O_2_) can increase the antithrombogenicity and cellular activity, making it a potential modification technique for bone regeneration (Uchida et al., 2014; Ribeiro et al., 2016). Wang et al. (2019) used a unique vacuum UV/O_3_ activation method to treat SF film, which improved the biocompatibility with BMSCs and osteogenesis *in vivo*. Kondyurin et al. (2018) applied plasma immersion ion implantation (PIII) to treat SF scaffold and receive a carbon-rich structure, which enhanced its interaction with both proteins and cells and exhibited significantly higher levels of cell adhesion and proliferation.

#### 4.3.2 Chemical modification

Considering the active groups of peptide chains, SF scaffolds have plenty of active modification sites, which allows different techniques for chemical modification, such as grafting copolymerization techniques and introduction of chemical agents (Murphy et al., 2008; Zhou et al., 2017; Zhou et al., 2018). Hardy et al. (2018) used a simple and rapid photochemical modification technique to initiate the polymerization and thus decorated the SF scaffolds with poly (acrylic acid) (PAA), poly (methacrylic acid) (PMAA), and poly (allylamine) (PAAm). The results showed that the PAA- and PMAA-functionalized SF scaffolds can be well-mineralized, indicating their potential in bone regeneration.

In term of 3D bioprinting of SF hydrogels, modification of vinyl-containing functional groups can help accelerate crosslinking process, especially methacrylate, which can make as-prepared glycidyl-methacrylate-modified SF (SF-MA) hydrogels photocurable. Barroso et al. studied the effect of the SF-MA solution pH on the properties of SF-MA hydrogels, where the SF-MA prepared at pH 5 exhibited better mechanical properties than that prepared in pH 7 and 8, and the hydrogel pH did not affect the good biocompatibility of as-prepared SF hydrogels.

#### 4.3.3 Surface functionalization

As for SF scaffolds, it is necessary to further improve osteogenesis especially in the repair of critical-sized or weight-bearing bone defects. Bioactive components and cells loaded on SF scaffolds have received increasing attention in bone regeneration. Different cytokines, drugs and pre-cultured cells can directly regulate cell behavior and promote bone regeneration.

During cell recruitment, proliferation and osteogenesis, cytokines play an important role (Lei et al., 2021; Wu et al., 2021). Loading cytokines on SF scaffolds is a promising method to direct cell differentiation and promote bone formation, including BMP-2 (Niu et al., 2017; Song et al., 2018), stromal-derived factor-1 (SDF-1) (Shen et al., 2016), VEGF (Besheli et al., 2018; Fitzpatrick et al., 2021), DFO (Wang et al., 2017; Ding et al., 2019) and so on. Fitzpatrick et al. (2021) generated multi-functionalized 3D-printed SF/HAP scaffolds loading BMP-2. VEGF and neural growth factor (NGF) to accelerate bone regeneration. The modulus of as-prepared scaffolds significantly increased and the expression of osteogenesis-related genes (Runx2, OPN, bone sialoprotein) was up-regulated by the synergistic enhancement of three factors.

**FIGURE 5:**
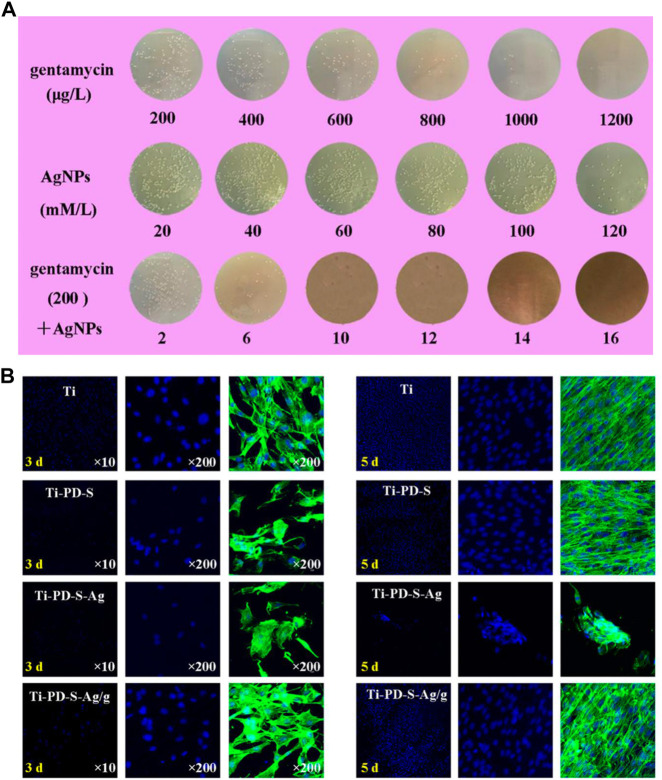
Antibacterial activities and biocompatibility of AgNPs/gentamicin-loaded SF scaffolds. **(A)** Quantitative evaluation of *S. aureus* cultured on different samples after 24 h **(B)** MC3T3 cell morphologies detected by fluorescent staining of FITC and DAPI (Zhou et al., 2017). Copyright 2017 American Chemical Society.

Infection and inflammation are significant challenges during bone regeneration. Drug-loading SF scaffolds seem a promising strategy to overcome these problems with application of antibacterial and anti-inflammatory drugs, such as gentamicin (Sharma et al., 2016), vancomycin (Besheli et al., 2017), Ag nanoparticles (AgNPs) (Zhou et al., 2017), dexamethasone (Moses and Mandal, 2022) and so on. Notably, combination of AgNPs and conventional antibiotics can achieve stronger antimicrobial performance and thus promote bone regeneration. Zhou et al. (Zhou et al., 2017) loaded AgNPs/gentamicin on SF scaffolds with the assistance of polydopamine, where AgNPs and gentamicin effectively inhibited adhesion and proliferation of *S. aureus*, and improved cell adhesion, proliferation and osteogenic differentiation simultaneously. (Figure 5). However, it still remains a challenge to avoid rapid drug releasing and obtain sustainable release and functionalization *in vivo*.

Besides, BMSCs and ADSCs are often seeded on SF scaffolds before implantation as bioactive components due to their differentiation potential. Plenty of researches indicated that pre-cultured BMSCs could effectively promote bone regeneration (Herrmann et al., 2015; Gao et al., 2016). Similarly, ADSCs exhibit anticipated osteogenic performance in different SF scaffolds, where ADSCs-loaded SF scaffolds can promote biomineralization and bone formation (Chen et al., 2016; Sartika et al., 2020). However, ADSCs alone are hard to achieve ideal differentiation towards osteoblasts without bioactive molecules or factors. It is essential to apply bioactive components as osteogenic inducers.

## 5 Conclusion and outlooks

SF scaffolds exhibit great potential in bone regeneration due to advantages of biocompatibility, biodegradability, mechanical strength and structural adjustability. With the rapid development of modern technology, a wide range of techniques has been employed to fabricate different types of SF scaffolds, such as films, porous scaffolds and hydrogels. For better functionalization and osteogenesis, SF scaffolds have been modified with plenty of components to maximize its mechanical and biological functional properties, such as angiogenesis, antibiosis, antiinflammation and cell-laden capacity. Based on recent technical advances in fabrication of SF scaffolds, researchers can better design and fabricate SF scaffolds according to practical demands of bone regeneration. Especially, with the development of 3D bioprinting, it has been feasible to achieve accurate shape and structures according the bone defects, which can accommodate living-cell patterning to mimic native bone tissues. These encouraging advancements have open up new opportunities in the application of SF scaffolds in bone regeneration, where controllable biodegradation and good mechanical properties are critically required.

Furthermore, SF hybrid scaffolds are able to simulate the natural bone microenvironment and promote osteogenesis with the help of well-designed structures, bioactive components and functional surface modification. However, there still lack sufficient studies on evaluation of SF scaffolds in big animal models such as dogs and pigs, which is necessary before future clinical applications. Meanwhile, considering their long-term close contact with tissues and the potential cytotoxicity and immunogenicity related to degradation products, it is vital to resolve the concerns of long-term *in vivo* safety of SF scaffolds. Therefore, although various techniques have been developed, it still needs more deep investigations to achieve comprehensive understanding of SF scaffolds.

Notably, although SF scaffolds have been investigated for a long time, it still has not been approved by regulatory authorities for any clinical application in bone regeneration. The clinical translation of such a Class III medical device can be quite time and cost-consuming and the translation process from bench to bedside is still unfamiliar to most researchers. So far, no clinical trials of SF scaffolds for bone regeneration have been registered in ClinicalTrials.gov or reported. Taken together, to promote better clinical translation of SF scaffolds for bone regeneration, more efforts should be carried out.

- More comprehensive investigations are required for deeper understanding of the degradation and clearing mechanisms of different SF scaffolds.
- More high-quality researches are in demand for further investigation on the degradation products of SF scaffolds to resolve concerns on the clinical use of SF scaffolds.
- More comprehensive translational researches for SF scaffolds are needed to make further steps to realize effective clinical translation, especially experiments in big animal models and clinical trials.

Overall, SF scaffolds are promising candidates to promote osteogenesis and have great potential in the field of medical devices for bone regeneration. This review intends to reveal the interaction between SF scaffolds and hosts’ cells and tissues, as well as up-to-date research status, which provides a deep and comprehensive insight into SF scaffolds for bone regeneration. It will help provide strong evidence to support the development and improvement of SF scaffolds for superior osteogenic performance.
